# Sequencing *PDX1* (insulin promoter factor 1) in 1788 UK individuals found 5% had a low frequency coding variant, but these variants are not associated with Type 2 diabetes

**DOI:** 10.1111/j.1464-5491.2011.03269.x

**Published:** 2011-06

**Authors:** E L Edghill, A Khamis, M N Weedon, M Walker, G A Hitman, M I McCarthy, K R Owen, S Ellard, A T Hattersley, T M Frayling

**Affiliations:** * Institute of Biomedical and Clinical Science, Genetics of Complex Traits, Peninsula College of Medicine and Dentistry, University of ExeterLondon; † Institute of Cellular Medicine, Newcastle UniversityLondon; ‡ Department of Diabetes and Metabolism, Barts and the London NHS Trust and School of Medicine and DentistryLondon; § Oxford Centre for Diabetes, Endocrinology and Metabolism, University of OxfordOxford, UK; ¶ Oxford NIHR Biomedical Research Centre, Churchill HospitalOxford, UK; ** Wellcome Trust Centre for Human Genetics, University of OxfordOxford, UK

**Keywords:** diabetes, genetics, polygenic, variants

## Abstract

**Aim:**

Genome-wide association studies have identified > 30 common variants associated with Type 2 diabetes (> 5% minor allele frequency). These variants have small effects on individual risk and do not account for a large proportion of the heritable component of the disease. Monogenic forms of diabetes are caused by mutations that occur in < 1:2000 individuals and follow strict patterns of inheritance. In contrast, the role of low frequency genetic variants (minor allele frequency 0.1–5%) in Type 2 diabetes is not known. The aim of this study was to assess the role of low frequency *PDX1* (also called IPF1) variants in Type 2 diabetes.

**Methods:**

We sequenced the coding and flanking intronic regions of *PDX1* in 910 patients with Type 2 diabetes and 878 control subjects.

**Results:**

We identified a total of 26 variants that occurred in 5.3% of individuals, 14 of which occurred once. Only D76N occurred in > 1%. We found no difference in carrier frequency between patients (5.7%) and control subjects (5.0%) (*P* = 0.46). There were also no differences between patients and control subjects when analyses were limited to subsets of variants. The strongest subset were those variants in the DNA binding domain where all five variants identified were only found in patients (*P* = 0.06).

**Conclusion:**

Approximately 5% of UK individuals carry a *PDX1* variant, but there is no evidence that these variants, either individually or cumulatively, predispose to Type 2 diabetes. Further studies will need to consider strategies to assess the role of multiple variants that occur in < 1 in 1000 individuals.

## Introduction

The study of the genetic component to complex diseases such as Type 2 diabetes has primarily focused on testing common variants. Genome-wide association studies focus on common single nucleotide polymorphisms, where common is usually defined as > 5% minor allele frequency. To date, there are over 30 replicated genome-wide association study case–control associations with Type 2 diabetes [1–3]. Despite the successful identification of many common variants involved in Type 2 diabetes, they explain only a fraction of the estimated genetic component. One possible explanation for this ‘missing heritability’ is that low frequency variants contribute substantially to the genetic risk of Type 2 diabetes. Most variants in the human genome are of low frequency (< 5%) and many more are < 1% frequency. Such low frequency variants are poorly captured by current genome-wide association study microarrays. Projects such as the 1000 Genomes Project are likely to reveal a much larger set of low frequency variants, but it remains a challenge to perform adequately powered association tests of such variants with human phenotypes. Few studies have tested comprehensively the role of low frequency variants in Type 2 diabetes, either genome wide or in the context of candidate genes. One study has tested the *WFS1* gene, but found no associations other than the well-replicated common variant (rs10010131) [4], and a second study tested part of the *HNF1A* gene [5] but did not identify any associated coding variants. Despite the challenges, there are several proof-of-principle examples that suggest sequencing strategies will identify low frequency variants involved in common human traits. These include the identification of rarer variants in the *IFIH1* gene that protect from Type 1 diabetes [6] and the low frequency variants in *NOD2* that have strong predisposing effects on Crohns disease [7]. Other approaches have shown that multiple low frequency coding variants in one or more genes accumulate at the tails of a population distribution for a continuous trait such as lipid levels [8] or blood pressure [9].

Mutations in the pancreatic and duodenal homeobox 1 (*PDX1/IPF1*) gene are a known cause of monogenic diabetes (OMIM 600733). *PDX1* is a key transcription factor involved in pancreatic development, islet hormone expression and the regulation of insulin in the mature B-cell. The importance of *PDX1* in pancreatic development is highlighted by the knockout mouse model, which has pancreatic agenesis [10]. This phenotype is mirrored in humans, where two different families have been described with pancreatic agenesis and neonatal diabetes attributable to recessive mutations [11,12]. Heterozygous mutation carriers have a later age of diabetes onset. *PDX1* has been previously studied as a candidate gene and in genome-wide association studies for Type 2 diabetes, but these approaches have been limited either to sequencing in small numbers of patients or common single nucleotide polymorphisms (genome-wide association studies) and there have been no robust associations with diabetes risk. In this study, we used an extensive re-sequencing approach to test the role in Type 2 diabetes of a comprehensive set of low frequency and rare *PDX1* variants.

## Subjects and methods

### Case–control cohort

The study population consisted of 910 patients with Type 2 diabetes. We selected patients diagnosed under 55 years, not insulin treated within the first year of diagnosis, with a median age of onset of 43 years (range 17–55 years) and a median BMI of 31 kg/m^2^ (range 18–58 kg/m^2^). The control population consisted of 878 normoglycaemic individuals, who were not known to have diabetes at time of blood collection, defined by a fasting blood glucose of < 5.5mmol/l and/or HbA_1c_ < 7% (< 53 mmol/ml). This population had a median age at sampling of 35 years (range 17–86 years) and a median BMI of 26 kg/m^2^ (range 17–49 kg/m^2^). All participants (patients and control subjects) were from the South West region of the UK and of European ancestry and came from four sample collections: the Exeter Family Study (control subjects), the Young Type 2 diabetes Study (YTYPE 2 DIABETES) and the Diabetes in Families Study (Warren2).

### Sequencing methods

We screened the two exons and approximately 50 bp of flanking sequence of the *PDX1* gene using bidirectional sequencing using standard conditions and following manufacturers’ protocols (primers available on request). Sequencing reactions were run on an ABI3730 capillary machine (Applied Biosystems, Warrington, UK). Sequencing was viewed in Mutation Surveyor (SoftGenetics, State College, PA, USA) (*PDX1* nucleotide reference NM 000209.3).

We used the bioinformatic tools, SIFT, PolyPhen and MutationTaster (http://blocks.fhcrc.org/sift/SIFT_dbSNP.html, http://genetics.bwh.harvard.edu/pph/, http://www.mutationtaster.org/)to predict the effect novel variants would have on the PDX1 protein (protein reference NP 000200.1).

### Statistical comparison

To compare the prevalence of individual variants and accumulations of variants in patients with Type 2 diabetes vs. control subjects, we used Fisher’s exact test. We had 80% power to detect variants that occurred in one control subject and seven patients with Type 2 diabetes at nominal levels of significance (*P* = 0.05)

## Results

### Molecular genetics

We sequenced 1788 individuals and identified 26 low frequency and rare variants in the *PDX1* gene. The detailed distribution of these variants within the cases and controls is shown in Table 1. Of these 26 variants, 22 were in the coding region, of which 17 altered the amino acid sequence, four were in the sequence immediately flanking the exons and 18 were novel (Table 1). Six of the variants were predicted likely to be deleterious in at least two bioinformatic programs, with three variants (P99H, E160V and R198C) predicted to be damaging by all three programs.

**Table 1:** *PDX1* rare variants identified in 910 patients with Type 2 diabetes and 878 control subjects

Position of change	AA change	Case subjects (*n* = 910)	Control subjects (*n* = 878)	Nucleotide change	SIFT/PolyPhen/MutationTaster†
*c.1-25insCTCCCGG		1	1	c.-25	NA
*2	N	3	3	c. 6 C > T	NA
*3	G > A	1	0	c. 8 G > C	−/+/−
18	C > R	0	1	c. 52 T > C	+/++/−
*33	P	2	1	c. 97 T > C/T > A	NA
33	P > T	6	5	c. 97 C > A	+/++/+
*55	G	1	3	c. 165 C > A	NA
76	D > N	12	11	c. 226 G > A	+/−/+
*95	P > Q	1	0	c. 284 C > A	−/−/+
*96	P > S	0	1	c. 286 C > T	−/++/−
*99	P > H	1	1	c. 296 C > A	+/++/+
*117	L > M	1	0	c. 349 C > A	+/−/+
140	A > T	2	0	c. 418 G > A	−/−/−
143	P > R	1	0	c. 428 C > G	+/+/+
*160	E > V	1	0	c. 479 A > T	+/++/+
197	R > H	1	0	c. 590 G > A	+/+/+
*198	R > C	2	0	c. 592 C > T	+/++/+
239	P > Q	8	10	c. 716 C > A	−/++/+
*242	P > L	4	3	c. 725 C > T	−/++/+
P243insPro(GCC)		1	0	c. 726 insGCC	NA
*245	G > R	0	1	c. 733 G > A	+/+/−
*250	P	0	2	c. 750 C > A	NA
*264	G	1	0	c. 792 C > T	NA
*IVS1 + 1 (c.406 + 1G > C)		1	0	c. 406 + 1 G > C	NA
*IVS2 – 8 (c.407 – 8G > T)		0	1	c. 407 – 8 G > T	NA
*IVS2 + 4 (c.846*4G > A)		1	0	c. 846 + 4 G > A	NA

* Novel variant.

† SIFT/PolyPhen/MutationTaster: − tolerated, + not tolerated (SIFT)/− benign, + possibly damaging, ++ probably damaging (PolyPhen)/− polymorphism, + disease causing (MutationTaster) (http://blocks.fhcrc.org/sift/SIFT_dbSNP.html, http://genetics.bwh.harvard.edu/pph/, http://www.mutationtaster.org/).

AA, amino acid; NA, not applicable.

### Association with diabetes

#### Individual rare variant analysis

For each of the 26 rare variants, there was no significant difference in frequency between patients and control subjects. Only one variant, D76N, occurred in more than 1% of individuals and was not associated with Type 2 diabetes (12 patients vs. 11 control subjects, *P* = 0.83).

#### Accumulation of low frequency variants in the PDX1 gene

We found that 5.3% of all individuals carried a variant in the *PDX1* gene, with no difference between patients (5.7%) and control subjects (5%) (*P* = 0.46). There were no differences between patients and control subjects when we carried out subgroup analysis comparing missense and frameshift variants (42 patients vs. 33 control subjects, *P* = 0.55), or variants that were unique to either patients or control subjects (13 vs. 5, *P* = 0.09), or unique to either patients or control subjects and in the coding region (8 vs. 3, *P* = 0.22). We considered those predicted to be deleterious by at least two bioinformatic programs and there was no significant difference (37 patients vs. 32 control subjects, *P* = 0.70). Finally, there were no differences when limiting the analyses to those variants in the DNA binding domain (137–203 amino acids), although all five variants identified in this region were in the patients (5 patients vs. 0 control subjects, *P* = 0.06).

In a secondary analysis, the 5.7% of patients carrying a variant were leaner [28.3 kg/m^2^ (18–44 kg/m^2^)] compared with patients not carrying a variant [31.3 kg/m^2^ (18–58 kg/m^2^)] (*P* = 0.002), but not diagnosed earlier (*P* = 0.15).

## Discussion

The identification of gene variants associated with Type 2 diabetes but not captured by current genome-wise association studies is important for two main reasons. First, such variants are likely to be of low frequency and may have appreciably greater effects on individual risk than the common variants typically identified by genome-wise association studies. Second, if such variants alter the coding sequence of genes, they could implicate the gene’s protein product in the aetiology of diabetes.

Our study represents one of few attempts to sequence the entire coding sequence of a known diabetes gene in more than a thousand individuals and test the individual and cumulative variants for association with Type 2 diabetes. Our results are analogous with those of Fawcett *et al*. [4], who showed that a large number of low frequency variants occur in the Wolfram syndrome gene, *WFS1*, but there is no evidence that these variants influence the risk of Type 2 diabetes. Eight per cent of UK individuals carry a low frequency variant in the *WFS1* gene and 5% carry a low frequency variant in the *PDX1* gene. Furthermore, the *PDX1* variant D76N has been widely studied, but the reproducibility of associations with Type 2 diabetes have varied [13–18]. Our data are in keeping with the recent meta-analysis of *PDX1* D76N case–control studies concluding that there is no association with Type 2 diabetes [19].

Together with the *WFS1* study, our results have a number of implications for the study of rarer genetic variation in diabetes. First, the results suggest that a large proportion of coding variants will be present at a low frequency, with all but one of the 26 variants we identified in *PDX1* in less than 1% of individuals. Such variants will need to confer odds ratios of 1.8 (for 1% frequency) to 4.5 (for 0.1% frequency) to be detectable at *P* = 5 × 10^−8^ in 10 000 patients with Type 2 diabetes and 10 000 control subjects. Second, 14 of these variants occurred only once in all 1788 individuals. This distribution of allele frequencies means that testing the cumulative effects of multiple low frequency coding variants could be used to potentially improve power. The power of such cumulative tests will depend on the proportion of variants that have a functional effect. Alternatively, studies of low frequency and rare variants should consider tracking variants through families and performing tests of linkage with reduced penetrance. The analysis of *PDX1* in further samples, and possibly functional studies, will strengthen the evidence for or against the role of coding variants specific to the DNA-binding domain of *PDX1*, where we found variants in five individuals with diabetes and none in control subjects. Finally, genome-wide analysis of low frequency variants in large sample populations may provide additional insights into the role of low frequency variants in Type 2 diabetes.

In conclusion, our study has shown that *PDX1* is an excellent candidate to capture low frequency variants; however, there is no evidence that these variants, either individually or cumulatively, predispose to Type 2 diabetes.

## Competing interests

Nothing to declare.

## Abbreviations

*PDX1*: pancreatic and duodenal homeobox 1 gene
